# Rapid review on GenAI in nursing education

**DOI:** 10.3389/frhs.2025.1725425

**Published:** 2026-01-15

**Authors:** Laura Hinsche, Martina Hasseler, Tim Tischendorf, Tom Schaal

**Affiliations:** 1 Faculty of Healthcare, Ostfalia University of Applied Sciences, Wolfsburg, Germany; 2 Westsachsische Hochschule Zwickau, Zwickau, Germany

**Keywords:** artificial intelligence, digital competence, digital nursing, ethical reflection, nursing education

## Abstract

**Backround:**

The use of generative AI, as represented by ChatGPT, holds promising potential for nursing education. This manifests itself in various areas, including personalized learning, simulation training and teaching process support. However, its integration requires careful consideration of ethical implications, adaptation of curricula and a high level of digital competence on the part of teachers. Only in this way can potential risks, such as the distortion of knowledge, bias and educational inequalities, be avoided.

**Methodes:**

Relevant publications were identified between 2019 and 2025 as part of a comprehensive literature search in the specialist databases PubMed, Embase, CINAHL and Scopus. The search was conducted using combined search terms that included the terms “generative AI”, “ChatGPT” and “nursing”. After removing duplicates and screening (PRISMA-guided), 140 full texts were analysed and divided into two publications. This rapid overview focuses on the topic of generative AI in nursing education.

**Results:**

As part of the analysis of the included studies, five thematic areas were identified, which were divided into the categories of nursing education, competence development and nursing skills, implementation possibilities, examination quality and ethical considerations, and evaluated. A key theme is the dual potential of this technology: it can enrich learning through features such as virtual tutors and improved exam preparation, but it also requires critical consideration of ethical issues such as plagiarism, data bias and the need for human oversight.

**Outlook:**

In this context, the conclusion emphasises the urgent need to adapt curricula and provide targeted further training for teachers so that GenAI can be used responsibly and effectively—rather than, as is often the case at present, by banning it altogether.

## Background

Recent literature explores the potential impact of artificial intelligence (AI) in nursing, including its anticipated role in transforming nursing care processes (1). AI is expected to enhance patient processes, treatment plans, and provide essential information for healthcare decisions, as well as aid in repetitive tasks and medication management (2, 3). Some researchers suggest that AI will automate routine tasks in healthcare, such as providing patient information and creating care plans (2). Studies such as that by Elmaoğlu et al. (4) demonstrate, using the example of health education for children, that ChatGPT has the potential to create interactive and more inclusive educational processes in healthcare. At the same time, however, key ethical and data protection challenges are to be expected (4). Sharma and Sharma (5) paint a very positive picture of generative AI (GenAI) and in particular ChatGPT and metaverse in nursing. ChatGPT can facilitate ongoing education and career advancement by tailoring learning paths to individual needs and interests. This ensures that nurses remain at the forefront of remote patient monitoring innovations, thereby upholding the highest standards of patient care. Overall, so Sharma & Sharma (5) ChatGPT has the potential to revolutionize nurse education. Educators are urged to proactively utilize ChatGPT and establish guidelines for its responsible implementation in classrooms. As nursing becomes increasingly technology-driven, it is crucial to educate students on scholarly integrity, patient safety, and ethical use of ChatGPT (6). With nurses comprising the largest contingent of healthcare workers globally, nurse educators have a unique opportunity to support students in practicing ethically and morally. Furthermore, nurses play a vital role in dispelling misinformation while caring for patients, making it essential for educators to raise awareness of resources and provide ChatGPT as a tool while acknowledging the controversies in nursing education and healthcare (6). For nurse educators and students, grasping the concepts of GenAI and AI in general is essential to leverage prompt engineering effectively for teaching, learning, and assessment purposes. For instance, educators could utilize it to develop lesson plans on various topics, create virtual simulation scenarios to educate students about patient issues, or design assessments and rubrics to evaluate knowledge and communication skills. Rani et al. (7) summarize following advantages for using ChatGPT in nursing education: students benefit from the accessibility and flexibility provided by ChatGPT in their learning journey. They can access information and refine clinical skills at their convenience, transcending time and place barriers. ChatGPT serves as a valuable resource by offering study materials, practice tests, and educational videos, enriching learning opportunities. It simplifies complex topics, rendering them comprehensible, and functions as a virtual tutor, tailoring learning experiences to individual needs (7). Incorporating AI tools into the classroom is imperative for equipping nursing students with the knowledge and ethical competencies essential for navigating the technology-driven healthcare environment (55). This integration fosters students' comprehension of AI's capabilities and limitations, crucial for fostering trust-based patient relationships in a technology-centric era. Ethical considerations, data privacy, and the necessity for comprehensive training in interpreting and utilizing AI-driven tools remain crucial areas to address in nursing education (Gagne et al., 2023). For nurse educators, a pivotal advancement entails a thorough evaluation of curricula to deliberately integrate AI into the classroom. Nurse educators acknowledge the emergence of ChatGPT and voice apprehensions regarding its potential misuse by students for outsourcing assessments and producing acceptable responses to prompts. The chatbots holds promise in enhancing learning and offering advantages to both educators and students (8). Nurse educators should thus be encouraged to explore ways that ChatGPT could be integrated into their curriculum through formative or summative assessments. At the same time, nursing students and educators must learn to deal with the limitations and critical aspects of ChatGPT and other GenAI tools, like significant ethical dilemmas. There is a potential for plagiarism and academic dishonesty if students excessively depend on ChatGPT-generated content without appropriate attribution and critical evaluation (7).

ChatGPT presents a double-edged sword for nursing education. On one hand, it provides easily accessible information, enabling nurses to stay current in the rapidly evolving healthcare landscape. It also supports educators by automating repetitive tasks through AI-powered tools and platforms. However, the integration of such technology requires careful ethical consideration. Key concerns include the risk of plagiarism and contract cheating, particularly when students submit AI-generated assignments without appropriate attribution. Moreover, unequal access to ChatGPT may deepen existing disparities in nursing education.

To address these challenges, Choi et al. (9) advocate for the responsible use of GenAI as a supplement—not a substitute—for traditional teaching approaches. They recommend that nurse educators receive targeted training to understand both the capabilities and limitations of ChatGPT, as well as its potential pitfalls. Such training can help educators implement strategies to detect and prevent misuse. While ethical issues must be acknowledged, the authors caution against outright bans in a world increasingly shaped by technology. With strategic implementation and critical oversight, tools like ChatGPT can become valuable assets in enhancing nursing education (9).

AI-powered clinical decision support systems should elucidate the rationale behind their suggestions, helping students grasp the underlying logic and evidence. Clearly outlining AI usage policies, standards, and decision-making procedures empowers students and reduces biases. Nurse educators have a vital role in fostering transparency by explaining how student and patient data is gathered, stored, and ethically managed. Implementing measures such as minimal data collection, anonymization, and secure data handling is crucial for safeguarding privacy and trust (Gagne et al., 2023).

Archibald (10) warns, that neglecting the presence and impact of ChatGPT in higher education can lead to immediate and multifaceted consequences, particularly in nursing and the health sciences. Failure to address its integration may jeopardize the integrity of student learning, as safeguards against AI-generated content would be lacking. Moreover, ignoring ChatGPT undermines the professional image of nursing and other health professions, potentially signaling a disregard for timely adaptation and relevance in professional education. Students would miss the opportunity to incorporate ChatGPT into their learning toolkit, depriving them of valuable resources. Additionally, avoiding critical analysis of ChatGPT's shortcomings impedes its potential development as a learning tool (10). Amid ongoing debates about GenAI in nursing education, this rapid review examines how GenAI is currently being applied in this field. Its goal is to provide an overview of both the state of research and the practical use of GenAI in nursing education.

Since GenAI has only been developing so rapidly for a few years and is increasingly finding its way into professional and everyday life, this study examines the current status and practical applications of generative AI in nursing education. It provides an overview of its use and highlights ethical and educational issues. The aim is to support the responsible use of GenAI tools in nursing education and to engage with current debates.

## Methods

A structured systematic literature review was conducted using specific search terms including “generative AI”, “GenAI”, “ChatGPT”, and “nursing” across several databases: PubMed, Embase, CINAHL, and Scopus. For each search term, corresponding MeSH terms were employed and combined using Boolean operators (Table 1). The review was restricted to publications from the last five years (2019–2025), highlighting the growing relevance of GenAI in healthcare. Inclusion criteria for this review were focused on the application of generative intelligence in professional nursing and the enhancement of nursing qualifications through AI. Exclusion criteria included machine learning, deep learning, and applications of AI in specific disease contexts. The detailed search strategies for each database are outlined in the subsequent overviews.

**Table 1:** Overview database research.

Database	Literature findings	Search terms
PubMed	433	[generative AI(tiab) OR gen AI(tiab) OR genAI(tiab) OR generative artificial intelligence(tiab) OR generative model*(tiab) OR generative language model*(tiab) OR LLM(tiab) OR large language model*(tiab) OR advanced language model*(tiab) OR deep learning model*(tiab) OR ChatGPT*(tiab) OR ChatGPT*(tiab) OR ChatGPT-3.5(tiab) OR ChatGPT-4(tiab) OR GPT-3.5(tiab) OR GPT-4(tiab) OR OpenAI(tiab) OR Bing Chat(tiab) OR Pi AI(tiab) OR Google Bard(tiab) OR AI-powered chatbot*(tiab) OR AI chatbot*(tiab) OR artificial intelligence-powered chatbot*(tiab) OR “Deep Learning”(mesh)] AND [nurse*(tiab) OR nursing(tiab) OR “Nurses”(mesh) OR “Nursing”(mesh) OR “Education, Nursing”(mesh)]
Embase	251	(“generative AI”:ab,ti OR “gen AI”:ab,ti OR genAI:ab,ti OR “generative artificial intelligence”:ab,ti OR “generative model*”:ab,ti OR “generative language model*”:ab,ti OR LLM:ab,ti OR “large language model*”:ab,ti OR “advanced language model*”:ab,ti OR “deep learning model*”:ab,ti OR ChatGPT*:ab,ti OR “ChatGPT*”:ab,ti OR ChatGPT-3.5:ab,ti OR ChatGPT-4:ab,ti OR GPT-3.5:ab,ti OR GPT-4:ab,ti OR OpenAI:ab,ti OR “Bing Chat”:ab,ti OR “Pi AI”:ab,ti OR “Google Bard”:ab,ti OR “AI-powered chatbot*”:ab,ti OR “AI chatbot*”:ab,ti OR “artificial intelligence-powered chatbot*”:ab,ti OR “generative artificial intelligence”/exp OR “ChatGPT”/de OR “deep learning”/de) AND (nurse*:ab,ti OR nursing:ab,ti OR “nurse”/exp OR “nursing”/exp OR “nursing education”/exp)
CINAHL	53	[TI (generative AI OR gen AI OR genAI OR generative artificial intelligence OR generative model* OR generative language model* OR LLM OR large language model* OR advanced language model* OR deep learning model* OR ChatGPT* OR ChatGPT* OR ChatGPT-3.5 OR ChatGPT-4 OR GPT-3.5 OR GPT-4 OR OpenAI OR Bing Chat OR Pi AI OR Google Bard OR AI-powered chatbot* OR AI chatbot* OR artificial intelligence-powered chatbot*) OR AB (generative AI OR gen AI OR genAI OR generative artificial intelligence OR generative model* OR generative language model* OR LLM OR large language model* OR advanced language model* OR deep learning model* OR ChatGPT* OR ChatGPT* OR ChatGPT-3.5 OR ChatGPT-4 OR GPT-3.5 OR GPT-4 OR OpenAI OR Bing Chat OR Pi AI OR Google Bard OR AI-powered chatbot* OR AI chatbot* OR artificial intelligence-powered chatbot*) OR “Deep Learning”(mesh)] AND (TI nurse* OR TI nursing OR AB nurse* OR AB nursing OR MH “Advanced Practice Registered Nurses+” OR MH “Nurse Practitioners+” OR MH “Pediatric Nurse Practitioners+” OR MH “Nursing Leaders+” OR MH “Nursing Staff, Hospital+” OR MH “Registered Nurses by Specialty+” OR MH “Education, Nursing+”)
Scopus	322	TITLE-ABS (“generative AI” OR “gen AI” OR genAI OR “generative artificial intelligence” OR “generative model*” OR “generative language model*” OR LLM OR “large language model*” OR “advanced language model*” OR “deep learning model*” OR ChatGPT* OR “ChatGPT*” OR ChatGPT-3.5 OR ChatGPT-4 OR GPT-3.5 OR GPT-4 OR OpenAI OR “Bing Chat” OR “Pi AI” OR “Google Bard” OR “AI-powered chatbot*” OR “AI chatbot*” OR “artificial intelligence-powered chatbot*”) AND TITLE-ABS (nurse* OR nursing)

The research covered the period from March 28, 2024, to September 1, 2025.

**Figure 1:**
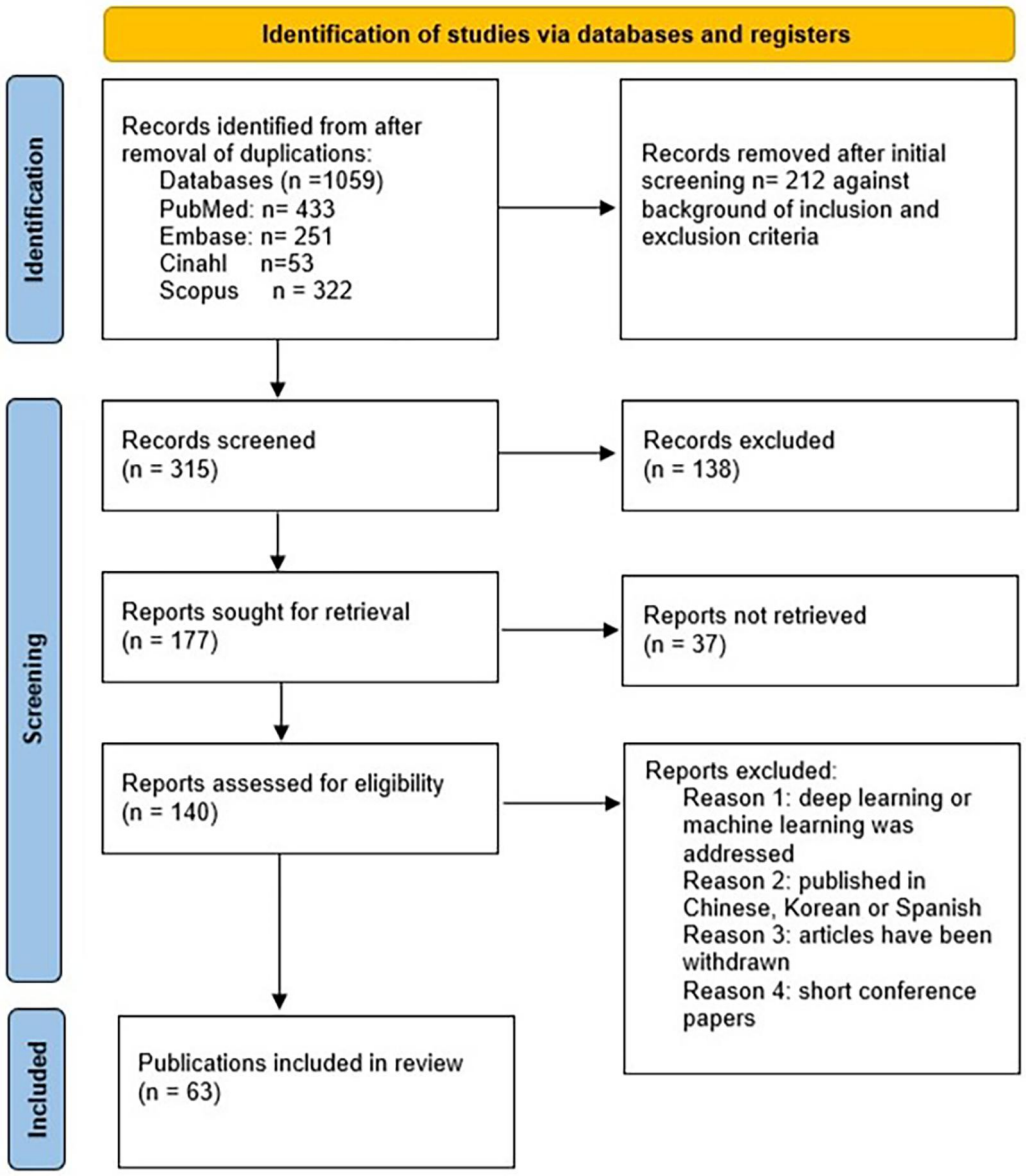
PRISMA flow diagram of the literature search and selection based on Moher 2009 (11).

In total, the search yielded 1,059 references, which were narrowed down to 315 referenes after removing duplicates. The PRISMA Flow Chart provides a detailed account of the literature search process and the selection criteria for the rapid review (Figure 1).

During the initial screening phase, 1,059 references were excluded based on the established inclusion and exclusion criteria. A subsequent, more detailed screening of 315 references resulted in the exclusion of 138 due to their irrelevance to the specified criteria. This process left 140 references for which full texts were obtained. However, 6 of these could not be located. Ultimately, 140 publications underwent a comprehensive analysis. Of these, 63 were excluded from the final analysis because they focused on machine learning, deep learning, specific diseases, were published in non-English languages, or were marked as retracted.

Early in the screening and analysis process, it became apparent that the literature on generative intelligence in nursing primarily addresses two distinct areas: its application in nursing education and its use in healthcare settings. To maintain clarity and focus due to the divergent nature of these topics, it was decided to divide the findings into two separate publications. This article, therefore, presents and discusses the results concerning GenAI in nursing education.

Preliminary investigation of the background and research context shows that researchers conduct few clinical studies on generative intelligence in nursing qualifications or healthcare in general. As the application of generative intelligence in healthcare is still in its early stages, this overview does not include a quality assessment of the publications. Instead, an integrated and narratively designed quick overview is provided, focusing on the role of GenAI in nursing education.

## Results

A total of five subject areas were identified in the analysis of the included studies. The content was divided into the subject areas of nursing education, competence development and nursing skills, implementation options, examination quality and ethical considerations. A core content table was created, divided into five categories.

### GenAI-Chatbots and AI simulation tools in nursing education

A recent empirical study by Jallad et al. (12) provides evidence to support the findings of this review by examining the use of AI tools in nursing education. A survey of 204 students revealed that AI applications for mobile devices, Microsoft PowerPoint functions and ChatGPT are already being used in practice. The authors identified significant correlations between frequency of use and parameters such as technology acceptance (TAM), self-efficacy in online learning (OLSE) and perceived system quality (ISSM) (12). Liu et al. (13) conducted a study on the perception and use of ChatGPT among nursing students at the West China School of Nursing. Nurse students primarily use ChatGPT to optimise their learning experience and for academic tasks, with a focus on term papers (13). However, in order to investigate integration and evaluate ChatGPT's performance in a Chinese nursing licence examination, Ni et al. (2024) examined, among other things, its accuracy compared to other language models. To prevent misuse, they emphasize the need for a regulatory framework and assessment methods. They conclude that ChatGPT is a promising tool for nursing education, but that it requires strict regulations for its implementation (56). With regard to the potential role of AI in education, particularly in relation to nursing curricula and specialised training programmes, considerable optimism is often expressed, although there are no specific application courses. According to the study conducted by Salama et al., the majority of nursing students at Palestinian universities were familiar with the subject of AI technology. However, it should be noted that 69.9% of respondents had not received any formal education or training in relation to ChatGPT. Despite this gap, 79% were in favour of its integration (14). Abujaber et al. (15) describe that GenAI-Chatbots can be used to develop personalized feedback, assistance, and direction for students. Tailored interactions with chatbots can notably enhance learning outcomes. Chang et al. (16) are conducting a study in which nursing students learn about vaccinations during pregnancy using GenAI-Chatbot compared to conventional teaching methods. A pre-post design with a mixed-method approach will be conducted. In the qualitative interviews, it becomes clear that the students feel more motivated to learn and engage with the topic with the chatbot approach (16). In another publication (17), three ways in which nursing students learn medical terminology are explored. The study evaluated the effectiveness of two innovative learning models in assisting nursing students in learning medical terminology concepts. It compared the impact of Termbot, a chatbot-based tool that offered gamified learning through crossword puzzles, and ChatGPT training to traditional textbook learning. Both AI-based technologies enhanced learning outcomes, with ChatGPT providing slight advantages and personalized learning based on student knowledge levels. The author highlights the potential of AI-powered educational tools in enhancing learning outcomes and emphasizes the need for further research to explore their effectiveness across diverse domains and larger student populations (17).

In practical skills education, GenAI can also be utilized. AI-driven simulations offer a safe and controlled environment for students to practice clinical decision-making and hone critical thinking skills. Sharpnack (18) emphasizes their ability to recreate diverse patient scenarios, fostering these crucial abilities (18). AI-generated patient narratives further enhance learning. Reed's (19) study demonstrates the positive impact of these narratives on student engagement and preparedness for simulations. These narratives, with both visuals and backstories, create a more immersive learning experience and promote empathetic care practices. However, the study also highlights the need to address potential biases in AI-generated content and ensure ethical use (20).

Streamlining scenario development is another benefit. Vaughn et al. (21) explored utilizing ChatGPT, a generative pre-trained transformer model, to create simulation scenarios. Faculty used ChatGPT to develop scenarios for various conditions, which were then reviewed by experts. While feedback was positive, the study revealed the need for expert review and refinement to ensure completeness and accuracy. ChatGPT serves as a valuable starting point, but additional information and adjustments are necessary for comprehensive and realistic scenarios (21).

Beyond simulations, AI holds promise for mitigating healthcare disparities. Chatbots and virtual environments can provide training and support for nurses in underserved regions with limited access to resources (5). Unlike traditional programs with geographic or financial barriers, these digital tools offer nurses in underprivileged communities the opportunity to receive quality training, fostering equitable access to education and ensuring they can deliver the best possible care to their patients (5).

### Competence development and nursing skills

Given the increasing significance of digital literacy as a fundamental competency in nursing informatics, prioritizing GenAI in course and program outcomes is imperative (8). As a result of this development and new demands on students, learning behaviour and competence development were examined in this rapid review.

GenAI serves as a personalized and self-learning tool, aiding students in enhancing their clinical judgment and overcoming language barriers and inequalities (6). Chang et al. (16) also compared traditional learning methods with a control group who were able to use ChatGPT for learning support on the topic of vaccinations during pregnancy. The students in the intervention group show a higher level of self-efficacy compared to the control group. The intervention group also achieve better results than the control group with regard to the Learning Achievements outcome (16).

ChatGPT and similar GenAI chatbots offer exciting possibilities for personalized learning in nursing education. Research by Abujaber et al. (15) highlights the effectiveness of chatbots tailored to individual student needs. Students receiving personalized interactions with chatbots showed improved performance compared to those with generic feedback. These AI-driven learning tools adapt to a student's learning pace, style, and preferences, providing targeted support for achieving educational goals (15). AI-powered simulations offer a safe and controlled environment for nursing students to hone their clinical skills. Sharpnack (18) emphasizes the advantages of these simulations, which recreate various patient scenarios. Students can practice clinical decision-making and develop critical thinking abilities through exposure to a range of simulated situations (18). The metaverse, a virtual reality space, holds promise for enhancing nursing education. Sharma and Sharma (5) discuss how metaverse training fosters collaboration and communication among nurses in a virtual setting. This virtual environment improves teamwork and decision-making skills, preparing nurses for effective remote patient monitoring. By equipping them to make informed decisions in complex patient scenarios, metaverse training ultimately leads to better patient care (5). The innovative virtual reality training program by Scott et al. (22) for novice nurses integrates 3D modelling, animation and language models to improve communication, history-taking and decision-making skills in controlled, authentic patient interactions. The use of technologies such as Unreal Engine and Meta Quest headsets, together with LLMs, enables dynamic, lifelike conversations with virtual patients. The aim is to better prepare students for clinical environments and boost their confidence (22).

Overall, advancements in AI offer a multitude of benefits for nursing education. From personalized learning with chatbots to advanced clinical simulations and metaverse training, AI tools can significantly enrich the learning experience and equip nurses with the necessary skills to thrive in the healthcare landscape. The study by Benfatah et al. (23) examines ChatGPT's role as a virtual patient in medical simulations, comparing its benefits to traditional patient simulations and identifying potential drawbacks. Participants interacted with ChatGPT individually in a simulation resembling real patient care, with interactions recorded for analysis. Students demonstrate adaptability in responding to virtual patient questions, suggesting their ability to tailor responses to specific patient needs (23).

In Reed's (19) study of using AI-generated images and narrated patient backstories to enhance preparation for nursing simulation, finds out that students experienced reduced simulation anxiety after viewing AI-generated backstories, indicating the potential of this approach to improve nursing simulation practices. The results highlight the positive impact of AI-generated visual narratives on student engagement and readiness for simulations, offering new avenues for innovative teaching methods and promoting empathetic care practices (19).

This integration fosters students' comprehension of AI's capabilities and limitations, crucial for fostering trust-based patient relationships in a technology-centric era. Moreover, teaching students to critically assess AI-generated content enhances their analytical abilities. Ethical considerations, data privacy, and the necessity for comprehensive training in interpreting and utilizing AI-driven tools remain crucial areas to address in nursing education (Gagne et al., 2023). At the same time, nursing students must learn to deal with the limitations and critical aspects of ChatGPT and other GenAI tools. When used critically and responsibly, it can enhance the learning process by providing access to a wealth of nursing-related information. Students can engage with the chatbot to obtain self-directed answers and learn effectively about various nursing topics and concepts.

### Implementation options

For the implementation of ChatGPT in nursing education Sharma and Sharma (5) believe, that ChatGPT and Metaverse offer promising avenues to enhance nurse education in remote patient monitoring, fostering abilities and confidence in managing such care scenarios. These technologies provide realistic and interactive simulations, boosting collaboration and decision-making among nurses. With its natural language processing capabilities, ChatGPT can simulate patient interactions closely resembling real-life situations, enabling nurses to refine their skills in a secure environment (5).

In Hsùs study, Termbot (Termbot, a chatbot-based tool accessible via a platform, offered gamified learning through crossword puzzles to enhance medical terminology understanding) and ChatGPT training were implementening in a learning scenario (17). The study by Higashitsuji et al. (24) shows that the use of ChatGPT in case-based learning (CBL) significantly reduces the time needed to create case vignettes without compromising the quality of learning discussions. Despite the lack of significant discrepancies in perceived learning quality, the study results point to a potentially efficiency-enhancing integration of GenAI into lesson planning (24).

Another way to application ChatGPT in nursing education is, having students develop educational materials for patients, and translating information into different languages can facilitate learning and application. ChatGPT could also assist students by improving their reading comprehension skills if English is not their native language or simplifying abstract or complex topics if they are struggling to grasp them through automated tutoring. For instance, one could ask for a simple and concrete example of how the adoption of a humanistic approach to nursing care compares to the traditional alternative. They could also ask for an example of nursing (8).

Gosak et al. (25) use a case study and ask ChatGPT which nursing diagnoses (NANDA-I) are present. They base the case on use Benner's theory (From Novice to Expert) to show at each level how the Prompts in ChatGPT can be formulated in order to gain knowledge appropriate to competence and level (26). The questions that are entered into ChatGPT are followed by questions of what source these nursing diagnoses are taken from. As a result of this study, ChatGPT has formulated nursing diagnoses that do not exist. In some cases, incorrect answers are given, it is observed that only one nursing diagnosis provided by ChatGPT aligned with the latest version of the North American Nursing Diagnosis Association—International (NANDA-I). However, ChatGPT correctly identified most of the health problems and presented the areas in which the case study patients had the most important health needs (25).

For nurse educators and students, grasping the concepts of GenAI and AI in general is essential to leverage prompt engineering effectively for teaching, learning, and assessment purposes. For instance, educators could utilize it to develop lesson plans on various topics, create virtual simulation scenarios to educate students about patient issues, or design assessments and rubrics to evaluate knowledge and communication skills (27). A cross-sectional study involving 95 nursing students shows a positive attitude towards the use of GenAI in the field of psychiatric nursing. The use of ChatGPT for completing tasks, creating case studies and understanding clinical case studies in the field of mental health was particularly associated with a significantly stronger positive perception of the learning effect (28).

Additionally, nursing students can employ prompt engineering across a multitude of GenAI tools to acquire and digest information on diverse subjects, formulate ideas for presentations or assessments, or enhance language and communication skills, among other benefits (27, 57). According to the qualitative study conducted by Gunawan et al. (29), Indonesian nursing students provide information about the diverse uses of ChatGPT. These include its use as a research aid, as a tool for self-reflection, and for case analysis (29). The relevance of prompt engineering as a fundamental key competence emphasize as an integral part of digital education in the context of nursing education (30). Simms (31) calls for the consistent integration of GenAI skills into the training of nursing staff as a fundamental professional competence. She emphasizes that curricula and teaching methods need to be adapted to enable students to critically evaluate AI-generated content, understand the technologies and use them responsibly (31).

### Examination quality

Some international studies are investigating whether and how GenAI, mostly using ChatGPT, is able to successfully answer the questions of national nursing exams or nursing degree programs.

A Swedish study by Christiansen et al. (32) explored ChatGPT's ability to complete a semester six nursing exam on palliative care. The exam focused on a case study of a young man with a brain tumor and his deteriorating condition. Questions tested knowledge of palliative care, symptom assessment, patient and family needs, treatment evaluation, and healthcare systems. All exam questions were uploaded to ChatGPT after students had completed the exam. The results were mixed. Some answers provided by ChatGPT were “excellent and well-reasoned” while others “lacked content or were outright incorrect” (32). Notably, the AI's responses displayed a more “American style” compared to the students' answers. This highlights both ChatGPT's potential and limitations in replicating human-like responses and raises concerns about academic integrity. The study found that ChatGPT could answer questions at various levels of complexity. If a student had copied the bot's answers and provided references, they potentially could have achieved a passing grade (32). This underscores the need for educators to consider AI's potential impact on assessment methods.

A Taiwanese study by Huang (33) investigated how ChatGPT performed on the Registered Nurse License Exam (RNLE) compared to human nursing students. The RNLE, with a notoriously low pass rate (18%–20% in 2022 compared to historical rates of 45%), requires answering 370–400 questions. Notably, ChatGPT achieved scores between 51.6 and 63.8 across four exam rounds. The study revealed both promise and limitations of ChatGPT. While demonstrating rapid response times and a basic understanding of nursing knowledge, ChatGPT's answers were inconsistent. This suggests potential for confusion or misinterpretations in complex scenarios, leading to varied explanations. Overall, the authors suggest ChatGPT cannot replace core nursing skills like clinical reasoning and judgment, particularly in complex situations. Although it accurately answered over 50% of the RNLE questions, further research is needed to determine its effectiveness in promoting higher-order thinking skills. Additionally, concerns regarding potential inaccuracies, plagiarism from misleading responses, and “hallucinations” generated by ChatGPT warrant caution when using this technology (33).

Studies suggest ChatGPT offers potential as a learning tool for nurses preparing for exams, but highlight the need for further development (34). A Taiwanese study by Su et al. (34) evaluated ChatGPT's performance on the National Nursing Licensing Exam, covering various nursing topics. While achieving an impressive overall accuracy of 80.75%, exceeding the passing mark, its performance varied across subjects. ChatGPT excelled in General Medicine (88.75%) but struggled with Basic Nursing (63.00%). Notably, it faced difficulties with complex scenarios and multiple-choice questions. Despite these limitations, the study found ChatGPT proficient in both lower-order (memorization, application) and higher-order (analysis, evaluation) thinking skills. However, a crucial concern emerged: 14.25% of ChatGPT's answers contradicted its explanations, lowering its effective accuracy to 74.00%. The authors suggest improvements for ChatGPT, including specialized training for nursing exams, better handling of uncertainties, incorporating external knowledge sources, and potentially utilizing multiple language models to enhance accuracy and reasoning (34).

However, research by Su et al. (3) emphasizes the need for further development in ChatGPT, particularly for handling complex questions and explanations. They propose areas for improvement: specialized training for nursing exams, improved handling of uncertainties in complex scenarios, integration of external knowledge sources and utilizing multiple language models collaboratively (34).

Overall, while ChatGPT offers promise for revolutionizing nursing education, ensuring reliable performance and incorporating critical thinking elements require further development (34). As AI technology advances, its potential to support nursing education and potentially assist with clinical decision-making will continue to evolve. Research suggests ChatGPT holds promise as a learning tool for nurses, but further development is necessary to ensure reliable performance (34). Studies examining its effectiveness in nursing exams highlight both potential and limitations. For instance, Su et al. (34) found that while ChatGPT achieved a good overall accuracy on the Taiwanese National Nursing Licensing Exam, its performance varied across subjects and required improvement in handling complex scenarios and explanations. Kaneda et al. (35) explored advancements between GPT-3.5 and GPT-4 in the context of the Japanese National Nursing Exam (JNNE). Notably, GPT-4 significantly outperformed GPT-3.5, exceeding the JNNE passing standard in both basic knowledge and scenario-based questions. This suggests GPT-4's potential to contribute to real-world clinical decision-making in Japan, although further research is needed to confirm its effectiveness in real-world settings (35).

The potential of AI extends beyond supporting student learning. Cox et al. (36) investigated AI's role in generating questions for the US NCLEX (National Council Licensure Examination) licensing exam, is a computer-based adaptive test that must be taken by nursing graduates in order to be licensed as a registered nurse in the United States and Canada. Over 800 nursing faculty members assessed AI-generated questions alongside human-generated ones. The findings revealed promise for AI-generated questions in terms of clarity, grammar, and clinical relevance. However, some questions lacked sufficient difficulty or contained unnecessary complexity. This highlights the importance of a human “review and refine” step after AI question generation.

ChatGPT offers exciting possibilities for nursing education, providing innovative tools for content development, creating assessments (36), and potentially even supporting clinical decision-making (34). However, ensuring reliable performance, incorporating critical thinking, and maintaining human oversight are crucial aspects of developing and integrating AI tools (34, 36).

According to the actually study by Kuribara et al. (37), the potential of large language models was also evaluated in the context of the Japanese national nursing examination. ChatGPT-4 and Microsoft Copilot achieved sufficiently high scores to pass the exam, demonstrating their potential as learning and exam aids. At the same time, LLMs show weaknesses, particularly in questions relating to legal or social security-specific content (37).

However, research by Su et al. (34) emphasizes the need for further development in ChatGPT, particularly for handling complex questions and explanations. Additionally, a study by Zong et al. (38) in China found that ChatGPT consistently failed national medical exams (2017–2021). While it performed well in some areas, its overall performance highlights limitations in handling complex medical knowledge. Despite limitations, AI offers exciting possibilities (38). A study by Cox et al. (36) investigated AI-generated questions for the US NCLEX exam. While promising in terms of clarity and relevance, some questions required human refinement. This underlines the importance of human oversight alongside AI tools.

Overall, while ChatGPT holds promise for nursing education, ensuring reliable performance, incorporating critical thinking elements, and maintaining human oversight remain crucial. As AI technology advances, its role in supporting learning and potentially clinical decision-making will likely continue to grow. A Japanese study by Taira et al. (39) explored ChatGPT's performance on national nursing exams. The exam covers a broad range of topics, including basic nursing skills and specialty areas. Notably, ChatGPT achieved a passing score in 2019 and consistently neared passing in subsequent years, suggesting its effectiveness in core nursing knowledge. However, its performance dipped slightly in later years, possibly due to limitations in training data (pre-2021) and increasing complexity of exam questions (39). The study emphasizes the potential for AI to assist nurses, but not replace their judgment. ChatGPT could be a valuable tool for tasks like analyzing patient data to support decision-making or providing resources for emotional patient support (39). However, continuous learning and adaptation are crucial for AI models in healthcare to maintain relevance (39).

### Ethical considerations

Ethical considerations, data privacy, and the necessity for comprehensive training in interpreting and utilizing AI-driven tools remain crucial areas to address in nursing education (Gagne et al., 2023). These include, for example, the questionable reliability and accuracy of ChatGPT's responses. Despite its ability to produce text resembling human language, concerns remain regarding factual errors and inconsistencies in the information provided. Additionally, ChatGPT may encounter difficulties in comprehending complex medical terminology, spatial and logical reasoning, and managing uncertainty. It lacks capabilities such as image processing, mathematical calculations, and demonstrating genuine emotions and thoughts. Furthermore, the rapidity with which ChatGPT operates raises the risk of excessive dependence on technology, potentially undermining critical thinking, creativity, and problem-solving skills (7). In a qualitative study, Shen et al. (40) show that the use of structured prompt engineering methods can significantly improve the accuracy and clinical context of AI-generated care plans. At the same time, the authors point out challenges arising from inaccurate AI responses, a lack of emotional sensitivity, and data protection issues. These findings illustrate that the responsible integration of GenAI requires not only technical but also ethical and educational safeguards (40).

There is a potential for plagiarism and academic dishonesty if students excessively depend on ChatGPT-generated content without appropriate attribution and critical evaluation. Additionally, there are apprehensions regarding biases inherent in the training data used for developing AI models like ChatGPT, potentially leading to biased or discriminatory responses (7). The pilot study by Bouriami et al. (41) provides in-depth insights into the concerns of nursing educators regarding the use of ChatGPT in a teaching context. In this context, both methodological-pedagogical and ethical issues are addressed, such as competence in dealing with AI, the necessary reflection on the limits of technology and possible risks, such as bias, data protection or excessive demands on teachers (41).

Choi et al. (9) discuss potential dangers for nursing students and nursing education, such as the impact of ChatGPT on students' communication and interactions with educators and peers. Students might resort to ChatGPT to produce written assignments, such as essays, and submit them as their own work, constituting contract cheating or plagiarism, which contravenes nursing professionalism standards. Furthermore, the use of ChatGPT may exacerbate equity issues in nurse education. Students with access to ChatGPT may gain an unfair advantage by generating high-quality assignments, leading to disparities in learning outcomes and opportunities compared to those without access to the chatbot (9).

Individuals deserve to understand how decisions that affect them are made. Yet, many AI systems, especially those driven by GenAI, lack transparency, leaving outcomes unexplained. Archibald and Clark (10) warn that neglecting the presence and impact of ChatGPT in higher education can lead to immediate and multifaceted consequences, particularly in nursing and the health sciences. Failure to address its integration may jeopardize the integrity of student learning, as safeguards against AI-generated content would be lacking. Moreover, ignoring ChatGPT undermines the professional image of nursing and other health professions, potentially signaling a disregard for timely adaptation and relevance in professional education (10). According to Şengül (42), nursing students show variance in their attitudes towards AI. These attitudes are associated with distortions in ethical decision-making behaviour. This leads to the conclusion that the introduction and use of GenAI requires didactic support in order to ensure the strengthening of ethical reflection skills. Furthermore, it should be noted that AI is not only a potentially useful tool, but can also influence ethical decision-making. The integration of GenAI into nursing education therefore only makes sense if it is accompanied by structured reflection and awareness-raising processes (42). Rodrigues and Cruz Correia (43) are equally critical of the unreflective implementation of AI in nursing education. They call for the development of an evaluation system to ensure the ethical values of nursing through AI, the development of strategies to reduce bias through AI, and the creation of a framework for data protection and data security to strengthen ethical principles (43).

However, Sharma and Sharma (5) point out that despite the potential benefits of integrating technologies like virtual reality (VR) simulations and ChatGPT into nursing education, several challenges need to be addressed. These include the high cost and time required for development and deployment, ensuring the accuracy and realism of VR scenarios, and addressing ethical concerns such as patient privacy. However, they also note limitations, such as the scenarios often lacking critical details like vital sign trends and specific symptoms, which are essential for realistic simulations. In conclusion, ChatGPT has potential as a tool for simulation scenario creation but needs expert review and refinement to ensure completeness and accuracy (5).

## Discussion

This rapid review on the question of how and in what way GenAI is used in nursing shows a variety of results. A global discussion surrounds integrating GenAI tools like ChatGPT into nursing education (8). These large language models (LLMs) offer a range of benefits. They can generate patient education materials and translate information for non-English speakers, promoting patient safety and understanding (8). ChatGPT excels in creating high-quality written content, making it ideal for tasks like paraphrasing, generating ideas, and structuring information (44). Furthermore, AI empowers educators by providing access to extensive databases and patient data for evidence-based decision-making, research, and informatics skills development (8). LLMs also significantly aid educators in creating course materials, assessments, and study guides, streamlining the learning process (8). Additionally, AI can generate patient scenarios for collaborative problem-solving in team-based learning environments (8). Also reaches its limits in simulation-based, clinically complex scenarios. GPT-4.0 lagged behind the students' performance in most areas, particularly in terms of response inconsistencies, hallucinations and an inadequate overall assessment of clinical situations. The use of GenAI in nursing education must therefore be carefully monitored (45).

Research suggests that ChatGPT, a GenAI chatbot, offers significant potential for enriching the learning journey of nurses. By providing tailored, interactive, and constructive learning opportunities, ChatGPT could empower students to actively build and integrate knowledge (16). The quantitative cross-sectional study by González-García et al. (46) shows that the use of ChatGPT was associated with a significant improvement in students' academic performance. Female students in particular attest to ChatGPT's high relevance for completing academic tasks (46). Its potential impact extends beyond education, with possibilities for influencing clinical nursing care in the future. Embedding ChatGPT into various nursing courses is recommended, along with assessments of its limitations and effectiveness using diverse methods (33). Evaluating ChatGPT's responses across different clinical scenarios and settings is vital for understanding its utility (33). The metaverse, an immersive learning platform, can be synergized with ChatGPT to create a holistic training experience for students, fostering comprehensive skill development (5).

ChatGPT is just the beginning of a continuously evolving technological landscape. Rather than resisting change, nursing educators are urged to adopt an “appreciative inquiry approach” towards emerging technologies (8). This includes fostering an understanding of how to effectively utilize AI and critically engage with it. Integrating competencies related to AI into entry-to-practice standards is crucial. These competencies will guide educators in curriculum development and equip students to effectively engage with and evaluate emerging technologies (8).

As Li et al. (47) show, nursing educators in different contexts demonstrate varying degrees of digital competence. Consequently, teachers should receive targeted support in the area of digital teaching competence when implementing ChatGPT (47). Nursing educators play a vital role by teaching students about ChatGPT's advantages and disadvantages, developing strategies for its meaningful integration into education, and promoting critical thinking skills through appropriate assignments (9). By adopting this forward-thinking approach, nursing education can leverage the power of AI tools like ChatGPT while ensuring students are well-equipped to navigate the evolving healthcare landscape. According to Simms (31), the consistent integration of GenAI literacy into nursing education is necessary as a fundamental professional competence for teachers to pass on knowledge about the use of AI in nursing (31).

Nursing educators play a vital role by teaching students about ChatGPT's advantages and disadvantages, developing strategies for its meaningful integration into education, and promoting critical thinking skills through appropriate assignments (9). By adopting this forward-thinking approach, nursing education can leverage the power of AI tools like ChatGPT while ensuring students are well-equipped to navigate the evolving healthcare landscape. According to Simms (31), the consistent integration of GenAI literacy into nursing education is necessary as a fundamental professional competence for teachers to pass on knowledge about the use of AI in nursing (31).

Studies show AI chatbots like ChatGPT can achieve good results on nursing exams in some countries. This suggests continuous improvement as AI is trained with better data. Additionally, involving nursing faculty in data training for AI tools like ChatGPT can ensure relevant nursing information is included. For simulations, AI holds promise despite potential inaccuracies requiring faculty revisions. AI-generated virtual cases could significantly support remote learning of clinical knowledge for nurses.

While ChatGPT offers exciting possibilities for nursing education, critical considerations and challenges need attention. For Academic Integrity, AI plagiarism detection tools may disadvantage non-native English speakers and be easily bypassed, compromising the authenticity of student work and assessment methods (48). Excessive dependence on ChatGPT could hinder critical thinking skills crucial for nurses. Additionally, students might inadvertently share confidential patient information with AI tools, jeopardizing patient safety and privacy (48). ChatGPT's vast data training raises concerns about plagiarism and copyright infringement. The developers, OpenAI, are facing legal battles regarding training data (48). ChatGPT might perpetuate societal biases present in its training data, leading to biased or discriminatory outputs. Transparency about training data is critical to identify and mitigate these biases (48). Ethical principles like patient privacy and confidentiality must be paramount when using ChatGPT in nursing education, research, or patient care (49).

Abujaber et al. (15) highlight ChatGPT's limitations, including unreliability, data inconsistency, limited knowledge, inability to understand emotions, and susceptibility to misinformation and algorithmic bias. Despite its advanced outputs, ChatGPT might generate inaccurate or misleading information, potentially impacting patient care (48). Furthermore, Zhang et al. (50) expand the discussion on the role of GenAI in examination didactics and emphasize potential risks with regard to academic integrity and distorted response patterns (50).

Effectively integrating ChatGPT in nursing education requires careful consideration of these ethical and practical challenges. For the future development and integration of GenAI in nursing education, such as ChatGPT or other tools, it seems suggestable to enhance nursing expertise in ChatGPT. It´s vital to improve ChatGPT's understanding of nursing concepts and practices by refining training data and incorporating nursing-specific resources. Continuous improvement is necessary to keep ChatGPT updated with the latest nursing research and technology, requiring ongoing evaluation and feedback from nursing stakeholders (51). Ethical considerations are paramount when using AI like ChatGPT in nursing education. Additionally, integrating ChatGPT with clinical skills training through simulations and real-time feedback is crucial. Furthermore, preparing nurses for interdisciplinary teams requires features that teach collaboration and communication with other healthcare professionals. Finally, fostering critical thinking and clinical decision-making involves incorporating complex patient scenarios and promoting evidence-based practices. These advancements aim to equip nursing students with strong critical thinking and clinical judgment (51). According to the study by Bouriami et al. (41), the successful use of ChatGPT in nursing education depends on the attitude and reflective skills of the teachers. They recognise the potential for active learning settings, but also express ethical concerns. The authors emphasize that critical engagement with GenAI by educational staff is central to responsible implementation (41). Sharifi et al. (52) take a critical stance on the use of ChatGPT in nursing education. They caution that uncritical application may hinder the development of clinical judgment and reduce direct interaction with instructors. To counter these risks, Sharifi Kelarijani et al. (52), recommend establishing ethical-didactic guidelines and providing students with structured opportunities for reflection (52).

However, one aspect whose importance has been underestimated until now is becoming increasingly relevant. This is the question of ownership and co-creation in the context of data, especially with regard to personalized learning models. Concepts such as the Global Patient Co-Owned Cloud (GPOC) call for greater patient involvement in data sovereignty. This opens up new perspectives for the development of high-quality artificial intelligence (AI) applications in the context of nursing education (53).

At the same time, the potential of personalized learning paths supported by artificial intelligence should be considered. There is a risk that existing strengths will be further developed, while weaker areas of competence will be neglected. To prevent the passive adoption of AI-generated suggestions, human mentoring and the promotion of critical reflection by teachers are of central importance.

## Limitations

This paper was designed as a rapid review, explicitly foregoing any claim to completeness of all available studies. In the field of generative AI, particularly in the context of nursing education, there is a wealth of new research providing relevant insights. Technological developments and the multitude of application scenarios make it difficult to provide a definitive assessment. Furthermore, only articles that were published or accessible at the time of the research were taken into account. Future systematic reviews could build on this basis and include a broader spectrum of literature and perspectives.

## Outlook

In today's healthcare landscape, AI models are rapidly becoming essential in healthcare, with their use expected to grow. Nurses and nurse educators must adapt by integrating AI into their practice and keep up with technological advancements and include them in nursing education. Though AI chatbots like ChatGPT have limitations, continuous development is crucial to enhance their effectiveness in nursing.

Research on AI chatbots in nursing is limited, necessitating further studies to explore their potential and establish effective training and support mechanisms. More data on the advantages and limitations of AI-supported chatbot technology will help nurses make informed decisions about its integration into practice. GenAI shows promise in facilitating self-directed learning and enabling personalized, adaptable nursing education. Future research should examine the impact of GenAI on nursing students' academic performance and ethical decision-making (Gagne et al., 2023). Long-term studies could track students exposed to GenAI, evaluating their ethical decision-making in clinical scenarios. Exploring effective methods for integrating cyberethics into nursing curricula is also crucial. Comparative assessments of teaching strategies, such as case-based learning, role-playing, and online modules, can help determine the best ways to prepare nursing students to use AI ethically and responsibly.

To address these challenges at universities and teaching organisations, clear guidelines and institutional policies on AI use in academic work are crucial. Universities should develop policies outlining the appropriate use of AI tools, their limitations, and the consequences of misuse. Comprehensive education on AI's proper use and potential risks is essential for nursing students and educators alike (48).

Nursing curricula should embed critical thinking and ethical reasoning about AI, using case studies and discussions on responsible application (48). While AI can support administrative tasks, educators must prioritize teaching core nursing competencies. Research by Seo and Kim (54) highlights that GenAI is largely viewed as an opportunity in nursing education, yet systematic analysis of internal strengths, weaknesses, and institutional frameworks remains lacking. Addressing these gaps is essential to ensure the sustainable integration of AI into education and training (54).

Finally, the invaluable insights nurses offer into patient care highlight the importance of exploring their perspectives on emerging healthcare technologies to gauge their influence on both their professional practice and personal well-being and on the further development of GenAI and tools like ChatGPT.
