# Supplementary material for: Rapid review on GenAI in nursing education
Source: Front Health Serv. 2026 Jan 15;5:1725425. doi: 10.3389/frhs.2025.1725425 (PMC12852379; doi:10.3389/frhs.2025.1725425)
Supplement: Supplementary file 1 [file Datasheet1.pdf]

STUDY	TOPIC	METHOD	FINDINGS IN RELATION TO THE RESEARCH QUESTION
GEN AI-CHATBOTS AND AI SIMULATION TOOLS IN NURSING EDUCATION			
Jallad, S. T., Alsaqer, K., Albadareen, B. I., & Al-Maghaireh, D. (2024). Artificial intelligence tools utilized in nursing education: Incidence and associated factors. Nurse Education Today, 142, 106355. https://doi.org/10.1016/j.nedt.2024.106355	Use of AI tools (e.g., ChatGPT, PowerPoint AI) in nursing education and influencing factors	Descriptive cross-sectional survey among nursing students in Palestine; based on Technology Acceptance Model (TAM)	Nursing students already use various Gen-AI tools (e.g., ChatGPT) in their learning process. Key influencing factors for usefulness and sustainability were perceived usefulness, ease of use, and institutional support. Findings highlight the need to integrate AI tools systematically into teaching strategies to enhance learning outcomes.
Liu, J., Wu, S., & Liu, S. (2024). Perception of ChatGPT by Nursing Undergraduates. In Studies in *Health Technology and Informatics*. DOI 10.3233/SHTI240271	ChatGPT awareness and usage among undergraduate nursing students	Quantitative cross-sectional study using a structured questionnaire (n=51)	Majority of students were aware of ChatGPT and used it primarily to support learning and complete assignments. The study highlights the potential of ChatGPT in nursing education and calls for further research on its educational effectiveness.
Salama, N., Bsharat, R., Alwawi, A., & Khlaif, Z. N. (2025). Knowledge, attitudes, and practices toward AI technology (ChatGPT) among nursing students at Palestinian universities. *BMC Nursing*, *24*(1). https://doi.org/10.1186/s12912-025-02913-4	Knowledge, attitudes, and practices of Palestinian nursing students regarding AI and ChatGPT	Cross-sectional KAP study among nursing students at Palestinian universities (quantitative survey)	High awareness and positive attitudes toward AI integration into curricula, but lack of formal training, missing curricula, and financial barriers hinder effective implementation. Highlights the gap between potential and preparedness in nursing education regarding Gen AI.
Abujaber, A. A., Abd-Alrazaq, A., Al-Qudimat, A. R., & Nashwan, A. J. (2023). A Strengths, Weaknesses, Opportunities, and Threats (SWOT) Analysis of ChatGPT Integration in	SWOT analysis of ChatGPT integration in nursing education	Narrative review using SWOT framework (Strengths, Weaknesses, Opportunities, Threats)	Highlights the potential of ChatGPT to enhance nursing education through accessibility, adaptability, and cost-efficiency. Emphasizes the need for collaboration among educators, policymakers, and developers to ensure responsible and effective implementation.

STUDY	TOPIC	METHOD	FINDINGS IN RELATION TO THE RESEARCH QUESTION
Nursing Education: A Narrative Review. *Cureus*, *15*(11), e48643. https://doi.org/10.7759/cureus.48643			
Ni Z, Peng R, Zheng X, Xie P. Embracing the future: Integrating ChatGPT into China's nursing education system. Int J Nurs Sci. 2024 Mar 7;11(2):295-299. Doi: 10.1016/j.ijnss.2024.03.006. PMID: 38707690; PMCID: PMC11064564.	Integration of ChatGPT in Chinese nursing education and licensure exam support	Quantitative comparison of ChatGPT vs. other LLMs on China's National Nursing Licensing Exam; discussion of educational implications	Demonstrates ChatGPT's superior performance in nursing exams, emphasizing its potential to enhance learning outcomes. Calls for targeted regulatory frameworks and adapted assessment strategies to maximize benefits and minimize risks in educational settings.
Chang, C.-Y.Hwang, G.-J., & Gau, M.-L. (2022). Promoting students' learning achievement and self-efficacy: A mobile chatbot approach for nursing training. *British Journal of Educational Technology*, *53*(1), 171–188. https://doi.org/10.1111/bjet.13158	Mobile Chatbot vs. Lecture-Based Learning in Nursing Education (Vaccination in Obstetrics)	Experimental study comparing chatbot-based learning with traditional lecture format	Chatbot group showed significantly better learning outcomes and self-efficacy. Highlights the effectiveness of AI-based, interactive, and personalized learning in enhancing critical thinking and decision-making in nursing education.
Hsu, M.-H. (2023). Mastering medical terminology with ChatGPT and Termbot. *Health Education Journal*, Article 00178969231197371. Advance online publication. https://doi.org/10.1177/00178969231197371	Use of ChatGPT and Termbot for learning medical terminology in nursing education	Exploratory study with control group (n=20) and two experimental groups (n=20 each) using ChatGPT or Termbot over 2 months	Both AI tools (ChatGPT and Termbot) significantly improved students' learning outcomes compared to textbook learning. Highlights potential of Gen AI tools as effective supplements in nursing education.
Sharpnack, P. A. (2024). Made Better by Chat GPT: Cultivating a Culture of Innovation in Nursing Education: Cultivating a Culture of Innovation in	Vision for integrating AI tools like ChatGPT in nursing education	Theoretical/conceptual article with literature-based discussion	Advocates for the early and ethical integration of AI tools (e.g., ChatGPT) into nursing curricula to enhance critical thinking, patient simulations, and care planning. Emphasizes the need for faculty training and curriculum development to embed Gen AI meaningfully into nursing education

STUDY	TOPIC	METHOD	FINDINGS IN RELATION TO THE RESEARCH QUESTION
Nursing Education. *Nursing Education Perspectives*, *45*(2), 67–68. https://doi.org/10.1097/01.NEP.0000000000001242			
Reed, J. M., & Dodson, T.M. (2023). Generative AI Backstories for Simulation Preparation. *Nurse Emulator.* Advance online publication. **https://doi.org/10.1097/NNE.0000000000001590**	Use of Gen AI-generated patient backstories for simulation in nursing education	Qualitative cross-sectional survey with content analysis	The use of Gen AI images for patient storytelling improved students' engagement, reduced simulation anxiety, and enhanced emotional connection and preparatory knowledge, indicating pedagogical value for simulation-based learning.
Vaughn, J., Ford, S. H., Scott, M., Jones, C., & Lewinski, A. (2024). Enhancing Healthcare Education: Leveraging ChatGPT for Innovative Simulation Scenarios. *Clinical Simulation in Nursing*, *87*, 101487. **https://doi.org/10.1016/j.ecns.2023.101487**	ChatGPT for generating nursing simulation scenarios	Development and expert evaluation of 5 AI-generated health simulations by 18 professionals	ChatGPT can significantly reduce time in creating simulations and provide structured drafts, but lacks consistent detail and realism, making expert review essential for educational use.
Sharma, M., & Sharma, S. (2023). A holistic approach to remote patient monitoring, fueled by ChatGPT and Metaverse technology: The future of nursing education. *Nurse Education Today*, *131*, 105972. **https://doi.org/10.1016/j.nedt.2023.105972**	Integration of ChatGPT and Metaverse in nursing education	Conceptual academic article	Highlights the potential of combining ChatGPT for simulated dialogues and Metaverse for immersive training to enhance remote monitoring skills in nursing students. Emphasizes the need for a balanced, ethical, and cost-conscious implementation.
COMPETENCE DEVELOPMENT AND NURSING SKILLS			

STUDY	TOPIC	METHOD	FINDINGS IN RELATION TO THE RESEARCH QUESTION
Castonguay, A., Farthing, P., Davies, S., Vogelsang, L., Kleib, M., Risling, T., & Green, N. (2023). Revolutionizing nursing education through Ai integration: A reflection on the disruptive impact of ChatGPT. *Nurse Education Today*, *129*, 105916. https://doi.org/10.1016/j.nedt.2023.105916	Impact of ChatGPT on competence development and digital skills in nursing education	Academic article (conceptual analysis)	Highlights ChatGPT's potential to enhance digital competence and critical thinking among nursing students. Emphasizes the need for responsible integration into curricula and collaboration with regulatory bodies to develop national AI competence frameworks in nursing.
Athilingam, P., & He, H.-G. (2024). ChatGPT in nursing education: opportunities and challenges. *Teaching and Learning in Nursing*, *19*(1), 97–101. https://doi.org/10.1016/j.teln.2023.11.004	Integration of ChatGPT in nursing education	Theoretical/discussion paper	Emphasizes ChatGPT's role in competence development as a learning assistant. Highlights the need for responsible use, educator/student training, and managing ethical concerns such as academic integrity and misinformation. Supports thoughtful integration rather than exclusion
Chang, C.-Y., Hwang, G.-J., & Gau, M.-L. (2022). Promoting students' learning achievement and self-efficacy: A mobile chatbot approach for nursing training. *British Journal of Educational Technology*, *53*(1), 171–188. https://doi.org/10.1111/bjet.13158	Use of mobile chatbot for improving learning outcomes and self-efficacy in nursing education	Experimental design with control group	Students using the chatbot showed significantly better learning outcomes and higher self-efficacy. The chatbot supported interactive, contextualized learning, fostering clinical decision-making and engagement.
Abujaber, A. A., Abd-Alrazaq, A., Al-Qudimat, A. R., & Nashwan, A. J. (2023). A Strengths, Weaknesses, Opportunities, and Threats (SWOT) Analysis of ChatGPT Integration in Nursing Education: A Narrative Review.	SWOT analysis of ChatGPT integration into nursing education	Narrative review based on literature analysis	Identifies accessibility, adaptability, consistency, and cost-effectiveness as key factors. Emphasizes collaborative efforts among educators, policymakers, and developers to address risks and optimize ChatGPT's educational potential.

STUDY	TOPIC	METHOD	FINDINGS IN RELATION TO THE RESEARCH QUESTION
Cureus, *15*(11), e48643. https://doi.org/10.7759/cureus.48643			
Sharma, M., & Sharma, S. (2023). A holistic approach to remote patient monitoring, fueled by ChatGPT and Metaverse technology: The future of nursing education. *Nurse Education Today*, *131*, 105972. https://doi.org/10.1016/j.nedt.2023.105972	Use of immersive technologies (ChatGPT & Metaverse) for developing nursing competencies in remote patient monitoring	Theoretical/conceptual article with literature-based discussion	Highlights the potential of ChatGPT for simulating patient communication and the Metaverse for realistic clinical training. Emphasizes a combined approach to improve technical and interpersonal skills in nursing, while addressing ethical concerns, cost, and the risk of overreliance on technology.
Sharpnack, P. A. (2024). Made Better by Chat GPT: Cultivating a Culture of Innovation in Nursing Education: Cultivating a Culture of Innovation in Nursing Education. *Nursing Education Perspectives*, *45*(2), 67–68. https://doi.org/10.1097/01.NEP.0000000000001242	Advocacy for integrating AI tools (e.g., ChatGPT) into nursing education	Commentary / Expert opinion	Emphasizes the importance of preparing nursing students to use AI responsibly by integrating AI-driven tools into simulations, care planning, and critical thinking development. Calls for investment in faculty training and curriculum development to ensure ethical and practical integration of AI technologies.
Scott, A., McCuaig, F., Lim, V., Watkins, W., Wang, J., & Strachan, G. (2024). Revolutionizing Nurse Practitioner Training: Integrating Virtual Reality and Large Language Models for Enhanced Clinical Education. In *Studies in Health Technology and Informatics*.	Integration of VR and Large Language Models in nursing education	Pilot project (preliminary development phase)	Aims to enhance traditional clinical training through immersive virtual simulations using LLMs and VR. Focuses on developing communication, history-taking, and clinical decision-making skills in a safe environment. Emphasizes the need for educational oversight and realistic interaction design.
Benfatah, M., Marfak, A., Saad, E., Hilali, A., Nejjari, C., & Youlyouz-Marfak, I. (2024). Assessing the efficacy	Attitudes toward ChatGPT and its	Qualitative study with thematic analysis	Students see ChatGPT as helpful but not entirely trustworthy. They emphasize that human aspects of nursing—such as empathy and touch—cannot be replaced by AI. Curriculum adaptations are needed

STUDY	TOPIC	METHOD	FINDINGS IN RELATION TO THE RESEARCH QUESTION
of ChatGPT as a virtual patient in nursing simulation training: A study on nursing students' experience. *Teaching and Learning in Nursing.* Advance online publication. https://doi.org/10.1016/j.teln.2024.02.005	integration in nursing education		to integrate AI while preserving core nursing values. Offers insights into Gen Z nursing students' views on technology in education.
Reed, J. M. (2023). Using Generative AI to Produce Images for Nursing Education. *Nurse Educator*, *48*(5), 246. https://doi.org/10.1097/NNE.0000000000001453	Application of AI-generated images in nursing education	Expert opinion	Highlights the educational value of generative AI tools like DALL·E 2 and Midjourney. Suggests that AI-generated images can enhance student engagement, emotional intelligence, and clinical judgment through visual reflection and dialogue. Encourages ethical integration into simulation, classroom, and clinical contexts.
Shin, H., Gagne, J. C. de, Kim, S. S., & Hong, M. (2024). The Impact of Artificial Intelligence-Assisted Learning on Nursing Students' Ethical Decision-making and Clinical Reasoning in Paediatric Care: A Quasi-Experimental Study. *CIN - Computers Informatics Nursing*, *42*(10), 704–711. https://doi.org/10.1097/CIN.0000000000001177	Ethical framework for Gen AI implementation in nursing education	Conceptual paper / Ethical analysis	Explores how ethical principles—autonomy, nonmaleficence, beneficence, justice, and explicability—can guide the responsible use of Gen AI. Highlights the need to integrate these principles into curricula and develop clear guidelines. Emphasizes the role of nurse educators in fostering critical thinking and ethical reflection. Suggests future research on long-term impacts of AI on ethics and learning.
Choi, E. P. H., Lee, J. J., Ho, M.-H., Kwok, J. Y. Y., & Lok, K. Y. W. (2023). Chatting or cheating? The impacts of ChatGPT and other artificial intelligence language models on nurse education. *Nurse Education Today*, *125*, 105796.	Ethical concerns and pedagogical implications of ChatGPT use in nursing education	Conceptual paper / Literature-based discussion	Discusses risks such as overreliance on AI, loss of critical thinking, and academic dishonesty. Recommends against banning ChatGPT; instead, it encourages training students in critical and responsible use to enhance self-directed learning and ethical awareness.

STUDY	TOPIC	METHOD	FINDINGS IN RELATION TO THE RESEARCH QUESTION
https://doi.org/10.1016/j.nedt.2023.105796			
IMPLEMENTATION OPTIONS			
Sharma, M., & Sharma, S. (2023). A holistic approach to remote patient monitoring, fueled by ChatGPT and Metaverse technology: The future of nursing education. *Nurse Education Today*, *131*, 105972. https://doi.org/10.1016/j.nedt.2023.105972	Use of immersive technologies (ChatGPT & Metaverse) for developing nursing competencies in remote patient monitoring	Theoretical/conceptual article with literature-based discussion	ChatGPT and Metaverse offer immersive and interactive training environments for remote patient monitoring. Their implementation can enhance communication and decision-making skills, but requires clear educational strategies, ethical guidelines, and curricular integration
Hsu, M.-H. (2023). Mastering medical terminology with ChatGPT and Termbot. *Health Education Journal*, Article 00178969231197371. Advance online publication. https://doi.org/10.1177/00178969231197371	Use of ChatGPT and Termbot for learning medical terminology in nursing education	Exploratory study with control group (n=20) and two experimental groups (n=20 each) using ChatGPT or Termbot over 2 months	Integration of ChatGPT and Termbot led to significantly improved post-test scores in medical terminology. The tools showed promise as effective supplementary learning aids, supporting the implementation of AI-driven methods in nursing education.
Higashitsuji, A., Otsuka, T., & Watanabe, K. (2025). Impact of ChatGPT on case creation efficiency and learning quality in case-based learning for undergraduate nursing students. *Teaching and Learning in Nursing*, *20*(1), e159-e166. https://doi.org/10.1016/j.teln.2024.10.002	Implementation of ChatGPT in case-based learning (CBL) for nursing education	Feasibility study with time tracking and evaluation of student discussions	The use of ChatGPT reduced case creation time by ~33% (from 106 to 71 minutes) without negatively affecting discussion quality. This suggests ChatGPT can improve instructional efficiency and support educators in CBL implementation.

STUDY	TOPIC	METHOD	FINDINGS IN RELATION TO THE RESEARCH QUESTION
Castonguay, A., Farthing, P., Davies, S., Vogelsang, L., Kleib, M., Risling, T., & Green, N. (2023). Revolutionizing nursing education through Ai integration: A reflection on the disruptive impact of ChatGPT. *Nurse Education Today*, *129*, 105916. https://doi.org/10.1016/j.nedt.2023.105916	Impact of ChatGPT on competence development and digital skills in nursing education	Academic article (conceptual analysis)	Practical implementation includes using ChatGPT for patient education material, language translation, reading support, and concept simplification—offering concrete ways to integrate Gen AI into curricula.
Gosak, L., Pruinelli, L., Topaz, M., & Štiglic, G. (2024). The ChatGPT effect and transforming nursing education with generative AI: Discussion paper. *Nurse Education in Practice*, *75*, 103888. https://doi.org/10.1016/j.nepr.2024.103888	Implementation of ChatGPT for nursing documentation training using NANDA-I	Case study analysis using Benner's theory ("From Novice to Expert")	ChatGPT supported documentation training by identifying relevant health problems, but often failed to match official NANDA-I diagnoses. Educators should use it as a learning aid, not a replacement for professional judgment.
Reed, J. M., Aterio, B., O'Lear, T., Coblenz, H., & Metz, T. (2023). AI Image-Generation as a Teaching Strategy in Nursing Education. *Jl. Of Interactive Learning Research (2023) 34(2), 369-399*, *34*(2), 369–399.	Generative AI in Image Creation for Nursing Education	Qualitative case study with nursing students using Midjourney to transform text into images reflecting their perception of nursing	Gen AI-based image generation supported students' emotional engagement and identity formation in nursing education. Despite technical and ethical challenges (e.g., gender bias, inaccuracy), its responsible implementation offers innovative opportunities for simulation, reflection, and assessment through prompt engineering.
Albikawi, Z. F., & Abuadas, M. H. (2025). Investigating the Impact of ChatGPT's Utilization on Psychiatric Mental Health Nursing Learning: A Student Perspective. *Universal Journal of Public Health*, *13*(3), 659–668.	ChatGPT use in psychiatric nursing education	Cross-sectional study with 95 nursing students in Jordan	Students showed a positive attitude towards using ChatGPT for learning tasks in psychiatric nursing. The tool enhanced engagement, supported understanding of mental health cases, and facilitated the creation of relevant scenarios. These findings suggest strong potential for targeted integration of Gen AI in mental health education.

STUDY	TOPIC	METHOD	FINDINGS IN RELATION TO THE RESEARCH QUESTION
https://doi.org/10.13189/ujph.2025.130314			
Gunawan, J., Aungsuroch, Y., & Montayre, J. (2024). ChatGPT integration within nursing education and its implications for nursing students: A systematic review and text network analysis. *Nurse Education Today*, *141*, 106323. https://doi.org/10.1016/j.nedt.2024.106323	Perceptions of Indonesian nursing students on ChatGPT use	Qualitative study (thematic analysis of student reflections)	Students used ChatGPT as a research support, for self-reflection, and for case analysis. While they found it helpful, they emphasized the irreplaceable role of empathy and human touch in nursing. The study suggests the need for curricular revision to integrate Gen AI while preserving core nursing values.
O'Connor, S., Peltonen, L.-M., Topaz, M., Chen, L.-Y. A., Michalowski, M., Ronquillo, C., Stiglic, G., Chu, C. H., Hui, V., & Denis-Lalonde, D. (2024). Prompt engineering when using generative AI in nursing education. *Nurse Education in Practice*, *74*, 103825. https://doi.org/10.1016/j.nepr.2023.103825	Integration of Prompt Engineering in nursing education	Editorial/Theoretical perspective introducing the PAIR framework	Highlights the need for prompt engineering as a key digital competence in nursing education. The PAIR (Problem, AI, Interaction, Reflection) framework offers a structured method for formulating prompts and critically evaluating AI outputs.
Simms, R. C. (2025). Generative artificial intelligence (AI) literacy in nursing education: A crucial call to action. *Nurse Education Today*, *146*, 106544. https://doi.org/10.1016/j.nedt.2024.106544	Gen AI as a core nursing competence	Theoretical/Conceptual paper	Emphasizes the need for students to critically assess AI-generated content and use it ethically and responsibly. Highlights curriculum adjustments, ethical implications (e.g., data privacy, bias), and the urgency of establishing global standards for AI education in nursing to align technological progress with nursing value

STUDY	TOPIC	METHOD	FINDINGS IN RELATION TO THE RESEARCH QUESTION
Examination quality			
Christiansen, M., Normark, L., & Swenne, C. L. (2023). Hur AI-verktyget ChatGPT klarar en hemtentamen i palliativ vård. *Högre Utbildning*, *13*(2), 56–62. https://doi.org/10.23865/hu.v13.5331	Evaluation of ChatGPT's performance on a nursing exam	Experimental case study: ChatGPT completed a real palliative care exam (16 questions) from Uppsala University	ChatGPT passed the exam with 28 out of 36 points, producing answers that were often longer and better structured than those of students. However, some responses lacked accuracy or contained factual errors. Subtle signs of AI authorship included an American discourse style and occasional grammar issues. The study highlights challenges in detecting AI-generated content and raises questions about academic integrity in assessment.
Huang, H. (2023). Performance of ChatGPT on Registered Nurse License Exam in Taiwan: A Descriptive Study. *Healthcare (Basel, Switzerland)*, *11*(21). https://doi.org/10.3390/healthcare11212855	Evaluation of ChatGPT's performance on Taiwan's RN Licensing Exam (RNLE)	Descriptive study assessing ChatGPT's results on 4 national nurse licensing exams (2022–2023)	ChatGPT scored between 51.6 and 63.75 points (passing twice). While it demonstrated basic nursing knowledge and rapid response times, it also generated inconsistent and occasionally misleading answers. The study emphasizes that ChatGPT cannot replace critical thinking or clinical judgment and highlights the need for careful integration and further research into its educational effectiveness.
Su, M.-C., Lin, L.-E., Lin, L.-H., & Chen, Y.-C. (2024). Assessing question characteristic influences on ChatGPT's performance and response-explanation consistency: Insights from Taiwan's Nursing Licensing Exam. *International Journal of Nursing Studies*, *153*, 104717. https://doi.org/10.1016/j.ijnurstu.2024.104717	Evaluation of ChatGPT's performance on the Taiwanese National Nursing Licensing Exam	Experimental study analyzing ChatGPT's answers to 400 real exam questions	ChatGPT achieved an overall accuracy of 80.75%, surpassing the passing threshold. However, its performance varied by subject (e.g., 88.75% in General Medicine, but only 63.0% in Basic Nursing) and showed significant weaknesses in handling complex multiple-choice questions and clinical scenarios. The authors recommend specialized training, improved uncertainty handling, integration of external sources, and use of multiple models for more reliable performance

STUDY	TOPIC	METHOD	FINDINGS IN RELATION TO THE RESEARCH QUESTION
Kaneda, Y., Takahashi, R., Kaneda, U., Akashima, S., Okita, H., Misaki, S., Yamashiro, A., Ozaki, A., & Tanimoto, T. (2023). Assessing the Performance of GPT-3.5 and GPT-4 on the 2023 Japanese Nursing Examination. *Cureus*, *15*(8), e42924. https://doi.org/10.7759/cureus.42924	Comparison of GPT-3.5 and GPT-4 performance on the Japanese National Nursing Exam (JNNE)	Comparative study using questions from the 112th JNNE to evaluate both GPT versions	GPT-4 significantly outperformed GPT-3.5, achieving a 79.7% accuracy rate and surpassing the national passing threshold. Study emphasizes the importance of specialized training and user responsibility due to the risk of hallucinated or inaccurate responses.
Cox, R. L., Hunt, K. L., & Hill, R. R. (2023). Comparative Analysis of NCLEX-RN Questions: A Duel Between ChatGPT and Human Expertise. *The Journal of Nursing Education*, *62*(12), 679–687. https://doi.org/10.3928/01484834-20231006-07	Comparison of AI-generated and educator-written NCLEX-RN exam questions	Quantitative and qualitative comparison using Likert scales, chi-square tests, and binomial tests across grammar, clarity, clinical relevance, and terminology	No significant differences were found in clarity, grammar, difficulty, or clinical relevance between AI and educator-created questions. Faculty preferred AI-generated items in 3 out of 4 cases with clear preference. The study concludes that AI can be a valuable support in question development, but faculty review remains essential to ensure quality and appropriateness.
Kuribara, T., Hirayama, K., & Hirata, K. (2025). Performance evaluation of large language models for the national nursing examination in Japan. *Digital Health*, *11*, 20552076251346571. https://doi.org/10.1177/20552076251346571	Evaluation of LLM performance (ChatGPT-3.5, ChatGPT-4, Microsoft Copilot) on the Japanese National Nursing Examination	Comparative analysis of 692 official nursing exam questions from the past 3 years	ChatGPT-4 and Microsoft Copilot scored high enough to pass in all years; ChatGPT-3.5 did not. Errors were concentrated in questions on health policy and social security, highlighting limitations of LLMs with country-specific legal/demographic content. The study emphasizes the need for nurse educators to critically assess AI outputs and remain aware of model biases and content gaps.
Zong, H., Li, J., Wu, E., Wu, R., Lu, J., & Shen, B. (2024). Performance of ChatGPT on Chinese national medical licensing examinations: A five-year examination evaluation study for physicians, pharmacists and	ChatGPT performance on Chinese national licensing exams in medicine, pharmacy,	Quantitative evaluation using direct prompting on NMLE, NPLE, and NNLE questions	ChatGPT failed to meet the accuracy threshold (≥0.6) across all three exams and years. While the model performed better in domains like clinical epidemiology, overall results highlight significant limitations in complex, domain-specific knowledge. The authors emphasize the need for higher-quality training data and stress the potential of LLMs in education, provided critical human oversight is maintained

STUDY	TOPIC	METHOD	FINDINGS IN RELATION TO THE RESEARCH QUESTION
nurses. *BMC Medical Education*, *24*(1), 143. https://doi.org/10.1186/s12909-024-05125-7	and nursing (2017–2021)		
Taira, K., Itaya, T., & Hanada, A. (2023). Performance of the Large Language Model ChatGPT on the National Nurse Examinations in Japan: Evaluation Study. *JMIR Nursing*, *6*, e47305. https://doi.org/10.2196/47305	Evaluation of ChatGPT-3.5 on Japanese National Nursing Examinations (2019–2023)	Performance analysis across five years of national nursing exam questions, focused on non-English and patient-centred contexts	ChatGPT achieved a passing score in 2019 and remained close to the threshold in later years, indicating stable performance. Weaknesses appeared in pharmacology and legal topics, reflecting potential gaps in non-English training data. The study suggests future versions could serve as supportive tools for decision-making and emotional care in nursing, while emphasizing the necessity of continuous refinement and human oversight.
ETHICAL CONSIDERATIONS			
Gagne, J. C. de, Hwang, H., & Jung, D. (2023). Cyberethics in nursing education: Ethical implications of artificial intelligence. *Nursing Ethics*, 9697330231201901. https://doi.org/10.1177/09697330231201901	Ethical integration of Gen AI in nursing education	Conceptual and ethical analysis of AI use in education, with a focus on Cyberethics principles	The study outlines five core ethical principles—autonomy, nonmaleficence, beneficence, justice, and explicability—as a framework for integrating AI into nursing education. Key concerns include data privacy, reliability of AI-generated content, and the need for thorough training in AI literacy. The authors advocate embedding ethical AI use into curricula and developing long-term strategies to ensure responsible adoption.
Rani, B., Srivastava, S. P., & Thakur, S. (2024). Nursing Education in the Age of Chat Generative Pre-Trained Transformer: Current Roles and Future Perspective. *Archives of Medicine and Health Sciences*. Advance online publication. https://doi.org/10.4103/amhs.amhs_208_23	Ethical concerns and human-centeredness in AI-supported nursing education	Narrative literature review and conceptual reflection	The article explores the dual potential of ChatGPT in nursing education, but also raises significant ethical concerns. These include risks of misinformation and overreliance on technology. The authors emphasise that AI cannot replace human educators when it comes to fostering critical thinking, ethical reasoning and empathetic care. They advocate the establishment of clear ethical guidelines and emphasise the importance of maintaining a human-centred approach in order to safeguard essential nursing values.

STUDY	TOPIC	METHOD	FINDINGS IN RELATION TO THE RESEARCH QUESTION
Shen, M., Shen, Y., Liu, F., & Jin, J. (2025). Prompts, privacy, and personalized learning: Integrating AI into nursing education-a qualitative study. *BMC Nursing*, *24*(1), 470. https://doi.org/10.1186/s12912-025-03115-8	Ethical and educational challenges of AI-generated care plans through prompt engineering	Qualitative study with third-year undergraduate nursing students	The study shows that structuring prompts can improve the clarity and clinical relevance of AI-generated plans, but also raises concerns about ethics, including lack of emotional awareness, potential data breaches and the need for human oversight. The authors advocate the development of ethical frameworks and educational safeguards to ensure the responsible integration of Gen AI, emphasising that technological tools should complement, rather than replace, core human competencies in nursing.
Bouriami, A., Takhdat, K., Barkatou, S., Chiki, H., Boussaa, S., & El Adib, A. R. (2025). Insights into nurse educators' use of ChatGPT in active teaching methods: A cross-sectional pilot study. *Educacion Medica*, *26*(2). https://doi.org/10.1016/j.edumed.2024.101006	Ethical concerns and attitudes of nursing educators towards ChatGPT use	Cross-sectional pilot study with nursing educators in Morocco	The study revealed that ChatGPT was used relatively infrequently by educators, despite their moderate level of knowledge. A notable concern, especially among male educators, was the risk of plagiarism and academic dishonesty. People were often indifferent or negative about using ChatGPT in teaching. The research shows that policies and training are needed to support responsible use of AI in nursing education.
Choi, E. P. H., Lee, J. J., Ho, M.-H., Kwok, J. Y. Y., & Lok, K. Y. W. (2023). Chatting or cheating? The impacts of ChatGPT and other artificial intelligence language models on nurse education. *Nurse Education Today*, *125*, 105796. https://doi.org/10.1016/j.nedt.2023.105796	Ethical risks of ChatGPT use in nursing education	Theoretical/discussion paper	The authors highlight several key ethical concerns, including students' overreliance on ChatGPT, which could prevent them from developing critical thinking and clinical decision-making skills. Issues raised include academic dishonesty (e.g. contract cheating), challenges in detecting AI-generated work, and potential inequities due to unequal access to AI tools. Rather than banning ChatGPT, the authors recommend teaching students responsible use to support self-directed learning and uphold ethical standards.
Archibald, M. M., & Clark, A. M. (2023). ChatGPT: What is it and how can nursing and health science education use it? *Journal of Advanced*	Ethical and strategic considerations of ChatGPT in nursing and health education	Editorial/commentary	The authors argue that generative AI like ChatGPT will profoundly impact health sciences education. They outline three possible institutional responses: avoidance, prohibition, and integration. Advocating for the integration approach, they highlight the need to balance the educational benefits of ChatGPT with concerns about academic integrity and data privacy. Ignoring the technology, they warn,

STUDY	TOPIC	METHOD	FINDINGS IN RELATION TO THE RESEARCH QUESTION
Nursing, *79*(10), 3648–3651. https://doi.org/10.1111/jan.15643			could compromise professional credibility and hinder responsive curriculum development in nursing education.
Sengul, T., Bilgic, S., Macit, B., Sevim, F., Alik, S., & Kirkland-Kyhn, H. (2025). Evaluation of nursing students' ethical decision-making biases and attitudes toward artificial intelligence in nursing education. *Nurse Education in Practice*, *86*, 104432. https://doi.org/10.1016/j.nepr.2025.104432	Ethical decision-making and AI attitudes among nursing students	Descriptive cross-sectional study (n=265)	Nursing students generally held positive attitudes toward AI, but questioned its ethical accuracy. A positive AI attitude was correlated with greater trust in ethical decision-making. Students frequently facing ethical dilemmas showed fewer cognitive biases. The study concludes that ethical reflection, critical thinking, and AI ethics must be embedded in nursing curricula to prepare students for patient-centred, ethically sound care.
Rodrigues, D., & Cruz-Correia, R. (2024). Large Language Models in Nursing Education: State-of-the-Art. In *Studies in Health Technology and Informatics*. https://www.scopus.com/inward/record.uri?eid=2-s2.0-85202007888&doi=10.3233%2FSHTI240584&partnerID=40&md5=def483c235db054680b26164abd546a5	Ethical integration of LLMs (e.g., ChatGPT) in nursing education	Literature review of 19 Sources	The study highlights both opportunities (e.g., critical thinking, clinical simulation) and ethical challenges (e.g., data privacy, AI bias, academic integrity). The authors call for the development of evaluation frameworks to safeguard ethical nursing values, mitigate algorithmic bias, and implement robust data protection strategies before integrating LLMs into nursing education.
However, Sharma & Sharma (2023) point out that despite the potential benefits of integrating technologies like virtual reality (VR) simulations and ChatGPT into nursing education, several challenges need to be addressed.	Integration of ChatGPT and Metaverse in remote patient monitoring training	Theoretical Exploration / conceptual paper	The study highlights the potential of ChatGPT and VR to provide immersive and personalized learning experiences in nursing education. However, it emphasizes ethical concerns such as patient privacy, high costs, and the need for expert oversight to ensure realistic and clinically accurate simulations. The authors advocate for a holistic approach combining technology with human-centred pedagogy.
